# AgTC and AgETL: open-source tools to enhance data collection and management for plant science research

**DOI:** 10.3389/fpls.2024.1265073

**Published:** 2024-02-21

**Authors:** Luis Vargas-Rojas, To-Chia Ting, Katherine M. Rainey, Matthew Reynolds, Diane R. Wang

**Affiliations:** ^1^Department of Agronomy, Purdue University, West Lafayette, IN, United States; ^2^Wheat Physiology Group, International Maize and Wheat Improvement Center (CIMMYT), Texcoco, Mexico

**Keywords:** data pipeline, extract-transform-load, database, data aggregation, data processing, plant phenotyping

## Abstract

Advancements in phenotyping technology have enabled plant science researchers to gather large volumes of information from their experiments, especially those that evaluate multiple genotypes. To fully leverage these complex and often heterogeneous data sets (i.e. those that differ in format and structure), scientists must invest considerable time in data processing, and data management has emerged as a considerable barrier for downstream application. Here, we propose a pipeline to enhance data collection, processing, and management from plant science studies comprising of two newly developed open-source programs. The first, called AgTC, is a series of programming functions that generates comma-separated values file templates to collect data in a standard format using either a lab-based computer or a mobile device. The second series of functions, AgETL, executes steps for an *Extract*-*Transform*-*Load* (ETL) data integration process where data are extracted from heterogeneously formatted files, transformed to meet standard criteria, and loaded into a database. There, data are stored and can be accessed for data analysis-related processes, including dynamic data visualization through web-based tools. Both AgTC and AgETL are flexible for application across plant science experiments without programming knowledge on the part of the domain scientist, and their functions are executed on Jupyter Notebook, a browser-based interactive development environment. Additionally, all parameters are easily customized from central configuration files written in the human-readable YAML format. Using three experiments from research laboratories in university and non-government organization (NGO) settings as test cases, we demonstrate the utility of AgTC and AgETL to streamline critical steps from data collection to analysis in the plant sciences.

## 1 Introduction

As the cost of genotyping continues to decrease, acquiring and managing data associated with plant phenotypes and environmental conditions have emerged as considerable limiting factors in plant science research. In response, technological advancements in data acquisition have been able to greatly increase the volume of data that researchers are able to collect from experiments (Eitzinger, 2021; Machwitz et al., 2021). Despite improvement in increasing the throughput of measurements, new instrumentation has not entirely replaced traditional methods; rather, they are often used to complement the repertoire of conventional methodologies employed by research groups, especially for experiments carried out under field conditions (Coppens et al., 2017; Crain et al., 2022). For instance, standard methods used at the International Maize and Wheat Improvement Center (CIMMYT) for plant phenotyping in their applied crop research programs include all of the following: traditional observation-based methods, high-throughput and low-cost phenotyping tools, and highly specialized equipment (Reynolds et al., 2020). The situation is similar for university-based research labs, where new instruments and techniques are continuously being tested and adopted, but complementary ground-reference measurements are still retained (e.g., Ting et al., 2023).

Given the diversity of measurements made by plant science research groups, labs currently experience several challenges related to the collection, processing, and management of data (e.g., protocols presented in Pask et al., 2012). First, many kinds of measurements are still recorded on paper. This is true not only for those collected by hand but also for measurements collected using electronic instruments that have limited memory, i.e. only storing a small number of observations. For example, the chlorophyll meter SPAD-502plus (Konica Minolta; Osaka, Japan) can save just 30 measurements in memory; for this reason, researchers still commonly record these data on paper (Mullan and Mullan, 2012). Newer versions of devices can sometimes enable greater data storage (e.g., the Chlorophyll Meter SPAD 502DL Plus with Data Logger can store up to 4,096 measurements). However, researchers often only have access to the older versions due to budget constraints that limit the upgrading of still-functional equipment. The second challenge concerns the heterogeneous nature of data files (i.e. those differing in format and structure), as measurements commonly collected in plant science research can originate from different instruments or methods. This creates issues in efficient data integration and management (Neveu et al., 2019). The final challenge lies in the storage and management of research data after they are integrated, which commonly rely on spreadsheet files on personal computers (Elsayed and Saleh, 2018) or with file storage cloud services using non-standard naming conventions and nested directories. This creates potential issues for sharing data in standardized ways with version control. Overall, these observations likely indicate that the data landscape for experiments carried out in plant science research domains has become increasingly complex. Improving the data pipeline from collection and processing to storage and management would help enhance data interpretation to ultimately enable new discoveries.

A data pipeline is a sequence of processes that begins with collection and includes extraction, transformation, aggregation, and validation, and is complete when data are loaded into a database for eventual analysis (Munappy et al., 2020). Even though data pipelines are designed to enhance research productivity, their successful implementation is often hindered by infrastructural and organizational challenges. Munappy et al. (2020) speculated that several human aspects underlie impediments to the adoption of these pipelines, including resistance to change, and development complexity.

In plant science research, numerous commercial and open-source tools for improving various steps in the data pipeline have been developed. For example, software applications have been made available for phenotyping in breeding programs to improve data collection in the field. These include Field Book, an open-source Android application that enables direct data entry with a user-friendly interface using experimental information loaded by users via files known as field files (Rife and Poland, 2014); Phenobook, an open-source web application for collaborative research (Crescente et al., 2017); and AgroFIMS, an open-source web tool that was initially developed as an analytics platform for breeding, whose current version has been expanded for data collection (Devare et al., 2021). The Integrated Breeding Platform, another example, is a commercial data management service for plant breeding programs that provides software, support, and services for breeding data pipelines (Malosetti et al., 2016). Breedbase is a web-based application that allows management of phenotyping data, stores genotypic information, and can perform analyses related to genomic prediction (Morales et al., 2022). Enterprise Breeding System is an open-source software for breeding programs that enables management of germplasm trials and nurseries as well as data management and analysis (CGIAR Excellence in Breeding Platform, 2022). More recently, PhytoOracle was released to provide a suite of tools that integrates open-source distributed computing frameworks for processing lettuce and sorghum phenotypic traits from RGB, thermal, PSII chlorophyll fluorescence, and 3D laser scanner datasets (Gonzalez et al., 2023). For a comprehensive recent review of digital tools developed for field-based plant data collection and management, we refer the reader to Dipta et al. (2023).

**Figure 1:**
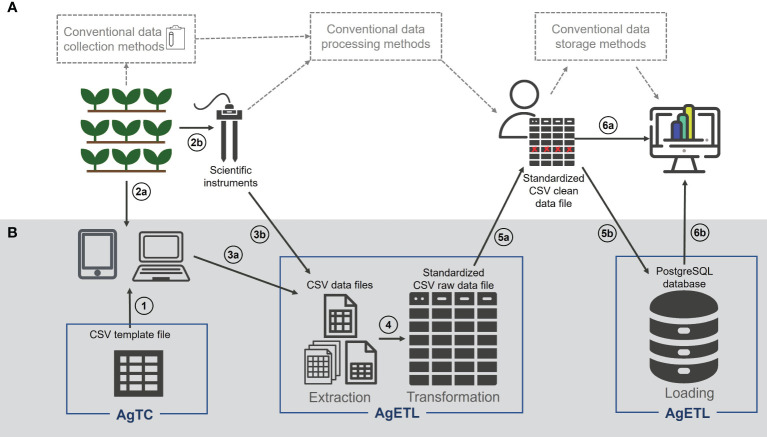
AgTC and AgETL can support plant science research from data collection to analysis. **(A)** Elements in the white background represent a typical series of steps taken in field- and controlled environment-based plant science research and **(B)** elements with grey background show how the processes of AgTC and AgETL can fit into within this overall framework. The proposed steps are numbered as follows: (1) Comma-separated value (CSV) template files for collecting data are created and uploaded to a computer or mobile device, where (2a) data are entered under experimental settings. (2b) AgTC-derived templates are not needed when data are collected using instruments that contain their data storage systems. Data that have been collected using the template from AgTC (3a) or downloaded from scientific instruments (3b) are next extracted using AgETL *Extract* functions and transformed using AgETL’s *Transform* process into a standard format in a single CSV file (4). (5a) Data quality assurance/quality control (QA/QC) processes are carried out outside the AgETL pipeline. (5b) When QA/QC is complete, data are ready to be loaded into a PostgreSQL database using the *Load* process of AgETL. CSV files containing clean data can be used directly for analysis (6a) or analyzed after it is downloaded from the database (6b).

Despite the repertoire of software described, current tools have several potential barriers to adoption in the broader plant science research community: they may be (1) commercial platforms; (2) open source or freely available but specialized for breeding application; (3) freely available but indicate that specialized IT knowledge is required; or (4) advertised as freely available but not actually available upon investigation. To address these gaps, we describe the development of two generic tools, called AgTC and AgETL, to enhance data collection and management in plant science research (**Figure 1**). These are alternatives for research groups that may not have the budget for a licensed plant-research database software platform or may require additional specialized IT knowledge to implement available free options. AgTC and AgETL also address the need for data collection and management tools to enhance data pipelines for plant science experiments that have objectives different from those of plant breeding programs (for which many tools are already available). For instance, they can be used in experiments where physiological traits or environmental factors are sampled at different time points with either traditional or modern techniques. The new AgETL tool is also amenable to help standardize data tables resulting from phenomics pipelines for final storage. Importantly, both AgTC and AgETL were designed based on extensive first-hand experiences of the primary tool developer as a field-based researcher who led data collection campaigns. Together, the two tools aim to reduce the time consumed on data processing and improve data storage to make downstream data analysis more efficient and accessible. While both tools work independently, they have a similar structure consisting of (1) an ipynb file executed on Jupyter Notebook, (2) function files containing Python functions that execute the steps for each tool, and (3) configuration files that contain user-specified parameters to run the functions; these are written in YAML, a data serialization language whose human-readable format functions correctly with Python (Ben-Kiki et al., 2021). Here, we demonstrate the utility of AgTC and AgETL in ongoing experiments on soybean, rice, and wheat carried out at Purdue University and at CIMMYT.

## 2 Methods

In this section, we describe the objectives and structure of the AgTC and AgETL Python functions along with implementation details and options for deployment. Experimental details from three test cases where data were collected using AgTC-generated templates are provided. Definitions for key terms mentioned throughout are first outlined below.

**Database**: A structured data collection stored and accessed electronically by a database management system (DBMS) such as MySQL, PostgreSQL, SQLite, Microsoft SQL Server, or Oracle Database. One of the characteristics of a DBMS is that it maintains data integrity; for instance, it does not permit storing different types of data in the same field or column or storing duplicated records based on the primary key, and data will persist as complete, accurate, and reliable.
**Dataframe**: A Python object where data are organized in tabular format (rows and columns). In contrast to a database that is stored on disk, a dataframe is not persistent because it is stored in memory (RAM).
**Database table**: A database object where data are stored in tabular format as records (rows) and fields (columns).
**Primary key:** Column of a database table that contains unique fields to enable the identification of single rows.

### 2.1 Agricultural data template creator

**Figure 2:**
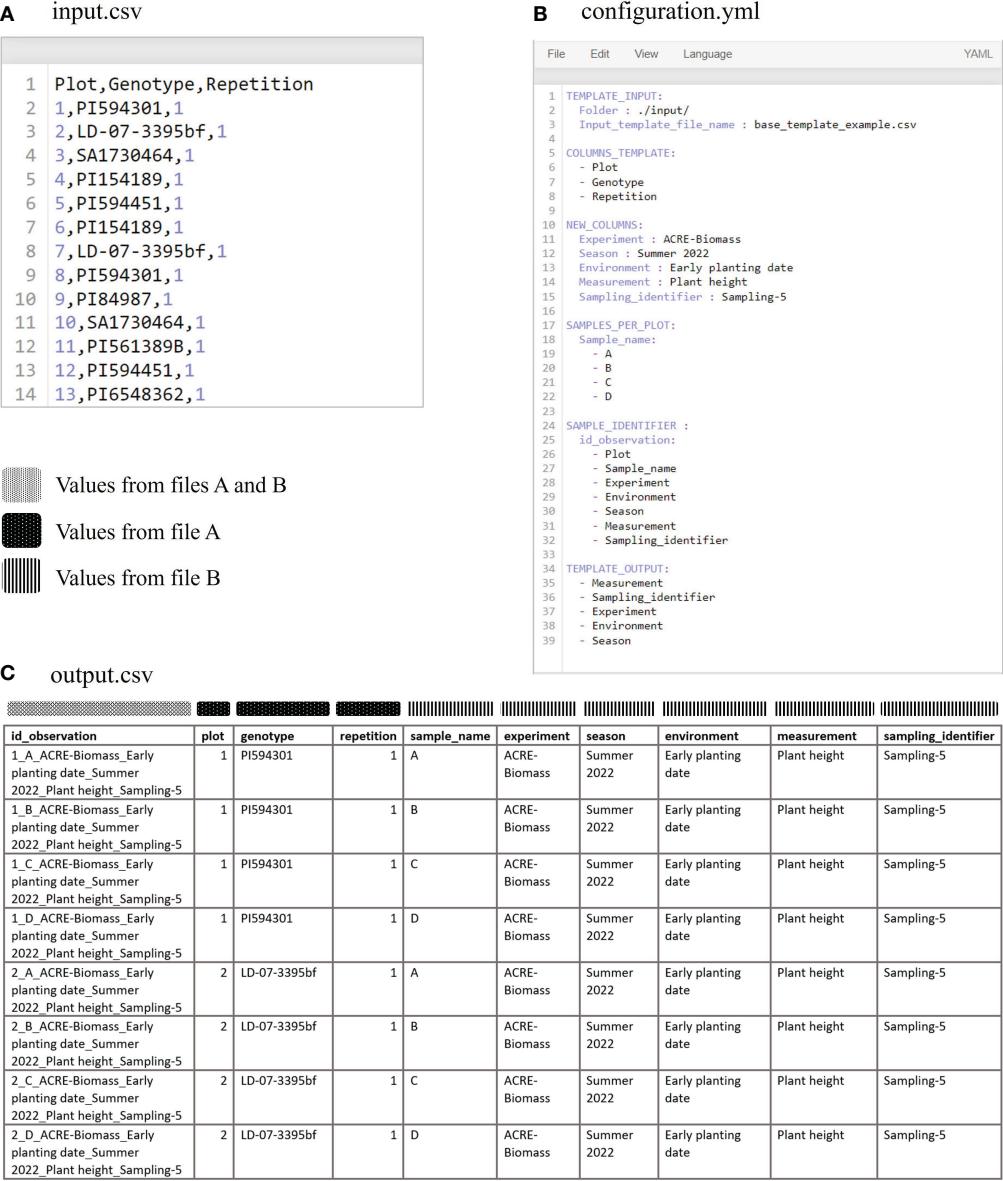
Input and output files of AgTC. Information from the input.csv file **(A)** and parameters from the configuration.yml file **(B)** are used to generate the columns found in the output template file **(C)**.

AgTC aims to standardize data collection for experiments conducted in field or controlled environment conditions. Its output is a CSV template file containing tabular meta-data related to the target observation in separate columns, such as crop species, experiment name, treatment, measurement name, unit of measurement, season, or other temporal designation, and one column that contains a unique identifier for each observation. These columns in the template are generated via two procedures. The first group of columns describes the experiment and is generated using basic metadata that are contained in an input CSV file. This may include a list of the experimental units (i.e., plots or pots), replications, genotype names, and any other information related to the experimental design. This set of information should be unique for each experimental unit. The second group of columns is generated using the parameters specified in the user-modified configuration YAML file, which also serves as an input file to AgTC. In contrast with the first group of columns, these columns, which are created using the YAML configuration file parameters, are repeated in all rows on a sequence basis. For clarification, **Figure 2** shows an example of an output template CSV created by AgTC and maps how information from input files is used to complete rows and columns in the output file.

All arguments (i.e., information passed into functions) for AgTC Python functions are taken directly from the user-specified configuration file; in this way, the user is able to add, delete, or modify parameters without the need to code in Python. The YAML configuration file is divided into six chunks of parameters known as *block collections*, where each block is identified with uppercase letters. A *block collection* may have *keys*, *values*, or *sequences* (**Table 1**). Considering that the block collection content can be modified to write variable names and content of rows, this enables the user to use controlled vocabularies and ontologies (Arnaud et al., 2020) or namings of crops, traits, and variables as recommended by the FAIR (Findable, Accessible, Interoperable, and Reusable) data principles (Devare et al., 2021). Once all the parameters are specified, the main.ipynb file can be executed on Jupyter Notebook without requiring the user to modify any line code. Since the template output is a CSV file, it can be opened by any spreadsheet software independent of operating system on a computer or mobile device to enter the observation values. Alternatively, the CSV file can be directly uploaded as a field file in the Field Book Android application (Rife and Poland, 2014) to facilitate data collection on the experiment in situ. The template created by AgTC fulfills Field Book's field file requirement of having a column containing unique observation identifiers and other columns that can be used to navigate within the application, such as plot number and treatment.

**Table 1:** The block collections of the AgTC configuration file.

Block collection	Description	Elements incorporated		
		keys	Values	Sequences
TEMPLATE_INPUT	Path and name of the base template file	U	U	
COLUMNS_TEMPLATE	Column names to take from the base template file			A D U
NEW_COLUMNS	Names and values for the new columns to add to the base template file	A D U	A D U	
SAMPLES_PER_PLOT	Number of repetitions of a measurement on the same experimental unit	U		A D U
SAMPLE_IDENTIFIER	Names of the columns with the values that will be used to create a unique identifier for each observation (row)	U		A D U
TEMPLATE_OUTPUT	Column names which values will be used to create the name for the new file			A D U

Letters indicate whether the user can add (A), delete (D), or update (U) the specific elements (*i.e.*, *keys*, *values*, and *sequences*) in each block collection.

### 2.2 Agricultural data extract, transform, and load framework

The objectives of the *Extract*, *Transform*, and *Load* (ETL) tool are to process CSV data files from different plant science experiments and aggregate them into a standard database table in a central repository. There, data are available to use for a variety of downstream analyses. The execution of functions in AgETL is divided into two Jupyter Notebook and configuration files. The first set of functions runs the *Extract* and *Transform* processes. This outputs a CSV file where the data from different source files have been aggregated and standardized into a single format. The second group of functions is used to load data into a single table in the database. The ETL functions are divided into these two separate groups (i.e., the *Extract* and *Transform* functions in one group and the *Load* functions in another) to enable users to carry out data quality control in between (**Figure 1**).

*Extract and Transform processes*: The *Extract* and *Transform* steps aim to merge different data files into a single and standard dataframe containing all necessary information to run subsequent analyses without needing extra information. These individual data files may be ones that were generated using template files from AgTC or those that were downloaded directly from scientific instruments. Files require a CSV extension and a grid-like format of rows and columns, with the first row containing column header names. Parameters are specified in a series of collection blocks found in the configuration file (**Table 2**), however, only the parameters within the FILES_TO_PROCESS collection block are required. The other parameters depend on the specific transformations that the data need for formatting into the final standardized dataframe. Therefore, the user can leave them empty if the data do not require transformation. All measurement values from different files are moved to the same column in the standardized dataframe, and they are differentiated from each other, adding the name and units of the variable in two different columns (see **Supplementary Figure 1**). The rest of the transformations are used to standardize row and column values, which are helpful for data aggregation. In this step, any unnecessary columns are also dropped. A CSV file containing all processed data in a tabular structure is exported at the end of the process.

**Table 2:** Block collections that are part of the two AgETL configuration files.

Group	Block collection	Description	Elements incorporated		
			keys	Values	Sequences
Extract-Transform	FILES_TO_PROCESS *	Path of the files to extract.	U	U	
	ADDITIONAL_INFORMATION_FILES	Path of the files that contain additional information to add	D U	D U	
	JOIN_FILES_COMMON_COLUMNS	Matching columns to merge the files to process and the additional information files			A D U
	COLUMNS_TO_DROP	Names of columns to drop			A D U
	UPDATE_COLUMN_NAMES	Update file column names	A D U	A D U	
	NEW_COLUMNS	New columns to add and the values to use for filling the rows	A D U	A D U	
	PRIMARY_KEY_COLUMN	Name of the column used as the primary key			U
	UPDATE_PRIMARY_KEY_VALUES	Values to replace parts of the string of the primary key	A D U	A D U	
	UPDATE_ROW_VALUES	Rows values to update	A D U	A D U	
	OUTPUT_FILE_NAME *	Name of new file for the standardized format data			U
Load	DATABASE_CREDENTIALS *	Credentials to establish the database connection	A D U	A D U	
	TABLE_NAME *	Name of the database table where data will be stored			U
	NEW_TABLE_COLUMNS	Names and data types for the columns to create if the table does not exist	A D U	A D U	
	NEW_COLUMNS_IF_TABLE_EXISTS	Names and data types of new columns to add if needed when the table already exists	A D U	A D U	
	FILE_TO_UPLOAD *	Path and name of the file to upload	U	U	
	PRIMARY_KEY_COLUMN *	Name of the column to use as the primary key			U

*Block collection that cannot be empty to run the code functions. Letters indicate permission that users have to add (A), delete (D), or update (U) each type of element (*i.e.*, *keys, values, sequences*) in the block collections.

*Load processes:* The objective of the *Load* processes is to upload the data into a database, with which users can interact to perform queries or carry out data analysis. Even if data reflect different species, experiments, seasons, variables, and sources, the *Load* functions are flexible to upload them in the same database table. This facilitates queries across experiments, researchers, species, etc., later. Those functions can also run structured query language (SQL) statements and open the database connection using six chunks of parameters in the configuration file. Out of the *block collections*, only four are required to load a new dataframe into the database table (**Table 2**). The other *block collections* are only required the first time a new table is created in the database or when a new column is appended to the table. The database is created in PostgreSQL, a DBMS based on a client-server architecture with a variant of the standard SQL as the query language (Herzog, 1998). Three functions create SQL statements to interact with the database. One of them allows creating a table (sql_statement_create_table_if_not_exist), another one inserts a new column in a preexisting table (sql_statement_add_column_if_table_exists), and another enables loading of the data into the table (insert_dataframe_to_database). Finally, execute_sql_statement is the function that executes the SQL statements. Additional SQL statements and commands found in the Jupyter Notebook allow the user to make changes and queries in the database. Since the *Load* operations are independent of the *Extract* and *Transform* steps, the process is amenable to uploading additional data files (e.g., metadata) and enables users to perform any SQL actions. For instance, the user can create tables and load data following an object-relational database model, which facilitates using Minimum Information about Plant Phenotyping Experiments (MIAPPE) standards (Papoutsoglou et al., 2020).

### 2.3 Implementation and deployment options

AgTC (source code available at https://github.com/DS4Ag/AgTC) and AgETL (source code available at https://github.com/DS4Ag/AgETL) are open-source and are available on GitHub. They can be executed under various versions of Jupyter. For example, they can run on a local server using a simple installation of JupyterLab or Jupyter Notebook, or they can run under environment management such as Conda, Mamba, or Pipenv. Another option to run these tools is on the cloud using a Jupyter Hub environment. The configuration file for the AgETL *Load* steps can establish a PostgreSQL database connection in a local host, such as installing it on a local development computer or using any of the standard service models of the database cloud service offered as Infrastructure as a Service, Platform as a Service, or Software as a Service (Jain and Mahajan, 2017). These options enable research labs to work with their institutional IT infrastructure to set up individual workflows or create their database using commercially available cloud services, including Amazon Web Services, Google Cloud Platform, or Microsoft Azure.

### 2.4 Test case 1: soybean evaluation under field conditions (USA)

As a first test case, we collected data on a soybean (*Glycine max*) experiment. This experiment evaluated 25 soybean genotypes with four repetitions and two treatments: early planting (planted on May 30, 2022) and late planting (planted on June 9, 2022). The trial was conducted at the Purdue University Agronomy Center for Research and Education (ACRE; 40° 28′ 20.5″ N 86° 59′ 32.3″ W) in West Lafayette, Indiana, USA. Various plant traits, such as height, width, growth stages, and photographs of a fully expanded trifoliate leave for each plot, were collected in the field using the Field Book application after creating templates using AgTC. These measurement campaigns occurred five times throughout the crop cycle, starting at late vegetative stages (V6) and finishing at approximately the R6 stage (full seed). In addition to data collected directly in the field, AgTC-generated CSV template files were used to record trifoliate dry weights on a computer in a lab setting. During the same measurement campaigns, volumetric soil water content (using HydroSense II; Campbell Scientific; UT, USA) and Leaf Area Index (LAI) (using the LAI-2200C Plant Canopy Analyzer; LI-COR Inc., NE, USA) were collected. In both these cases, data were initially stored in each of the devices’ internal memory. Thus, for soil moisture and LAI, there was no need to use the AgTC template. Finally, after the R8 growth stage (full maturity), plants were sampled and processed in the lab to obtain yield components (plants per meter, pods per plant, and seeds per pod). Data were entered into template files created by AgTC using a computer for these measurements.

### 2.5 Test case 2: wheat evaluation under field conditions (Mexico)

For our second test case, we collected data on wheat (*Triticum aestivum*) in an experiment established at CIMMYT’s research station, Campo Experimental Norman E. Borlaug (CENEB), located near Ciudad Obregon, Sonora, Mexico (27° 23′ 46″ N, 109° 55′ 42″ W). The trial evaluated a panel of 14 wheat genotypes with three repetitions under three environments: well-watered (WW), drought (DR), and high temperature (HT). Data were collected throughout the 2022 and 2023 crop growth seasons. Seedling emergence occurred in early December for the WW and DR treatments in both seasons. However, the HT trial was planted only for the 2022 season, with an emergence date in early March (**Supplementary Table 1**). Direct and proximal sensing measurements were made during the two crop seasons. There were four direct measurement sampling campaigns throughout each cycle. The first occurred before sowing, and the second was 40 days after seedling emergence. The third and fourth sampling campaigns were scheduled based on each genotype’s specific growth stages (GS) (Zadoks et al., 1974), with the third carried out 12 days after heading (GS54) and the fourth at physiological maturity (GS87). Proximal sensing measurements were made every week from canopy closure to GS87.

Template files created by AgTC were used to enter data for gravimetric soil water content, above-ground biomass, and yield components. Samples were first collected from the field and then processed in the laboratory. Growth stages, including seedling emergence (GS10), flag leaf sheath extending (GS41), GS54, and GS87, and one plant height measurement after GS87, were collected directly in the field using the AgTC template and the Field Book application. The proximal sensing data collected include chlorophyll content (using the SPAD-502 chlorophyll meter; Konica Minolta; Osaka, Japan), canopy temperature (using the Sixth Sense LT300 Infrared Thermometer; TTI Instruments; VT, USA), normalized difference vegetation index (using the GreenSeeker hand-held optical sensor; N-Tech Industries; CA, USA) and hyperspectral reflectance (using the ASD Field Spec 3; ASDInc., CO, USA).

### 2.6 Test case 3: rice evaluation in a controlled-environment facility (USA)

For our final test case, we collected data on cultivated Asian rice (*Oryza sativa*) in a growth chamber environment at Purdue University’s Ag Alumni Seed Phenotyping Facility (AAPF) (West Lafayette, Indiana, USA). Six genotypes were chosen based on their documented genetic information, constrained flowering dates, diverse geographical backgrounds, or potential genetic value (Rice Diversity Panels 1 and 2 from the USDA-ARS Dale Bumper National Rice Research Center, Stuttgart, Arkansas, Genetic Stocks Oryza Collection (www.ars.usda.gov/GSOR)) and raised for 82 days during Summer and Fall of 2022. The facility has two large growth chambers (Conviron^®^, Winnipeg, Canada) with a weight-based automated irrigation system (Bosman Van Zaal, Aalsmeer, The Netherlands), and both chambers were leveraged in this experiment; one had CO2 concentration at 700 ppm (high CO2 chamber) and the other at 415 ppm (ambient CO2 chamber). The rice plants were grown in pots under two levels of CO2 and two levels of drought. Each treatment had two replications with a total of 48 plants. According to the timing of drought, the experimental period could be divided into three timepoints: before drought (42-47 DAS, timepoint 1, TP1), during the mid of drought treatment (59-61 DAS, timepoint2 A, TP2-A), at the very end of drought (66-67 DAS, timepoint2 B, TP2-B) and upon recovering (77-82 DAS, timepoint3, TP3).

In this test case, AgTC was utilized to create Field Book field files to (1) collect photographs for later calculation of Specific Leaf Area (SLA), similar to the soybean test case (during TP2-B and TP3) and (2) record leaf water potential (LWP, MPa) measurements at 0800, 1400, and 1800 hr during TP2-B. For both types of measurements, the youngest fully expanded leaf from one plant was selected for each observation. For LWP, we used the Model 1000 pressure bomb (PMS Instrument Company, OR, USA).

## 3 Results and discussion

This section proposes a workflow using AgTC and AgETL to support plant science experiments from data collection to analysis. We describe the results of implementing the entire data pipeline in the two field experiments and utilizing AgTC in all three experiments.

### 3.1 Proposed data pipeline

**Figure 3:**
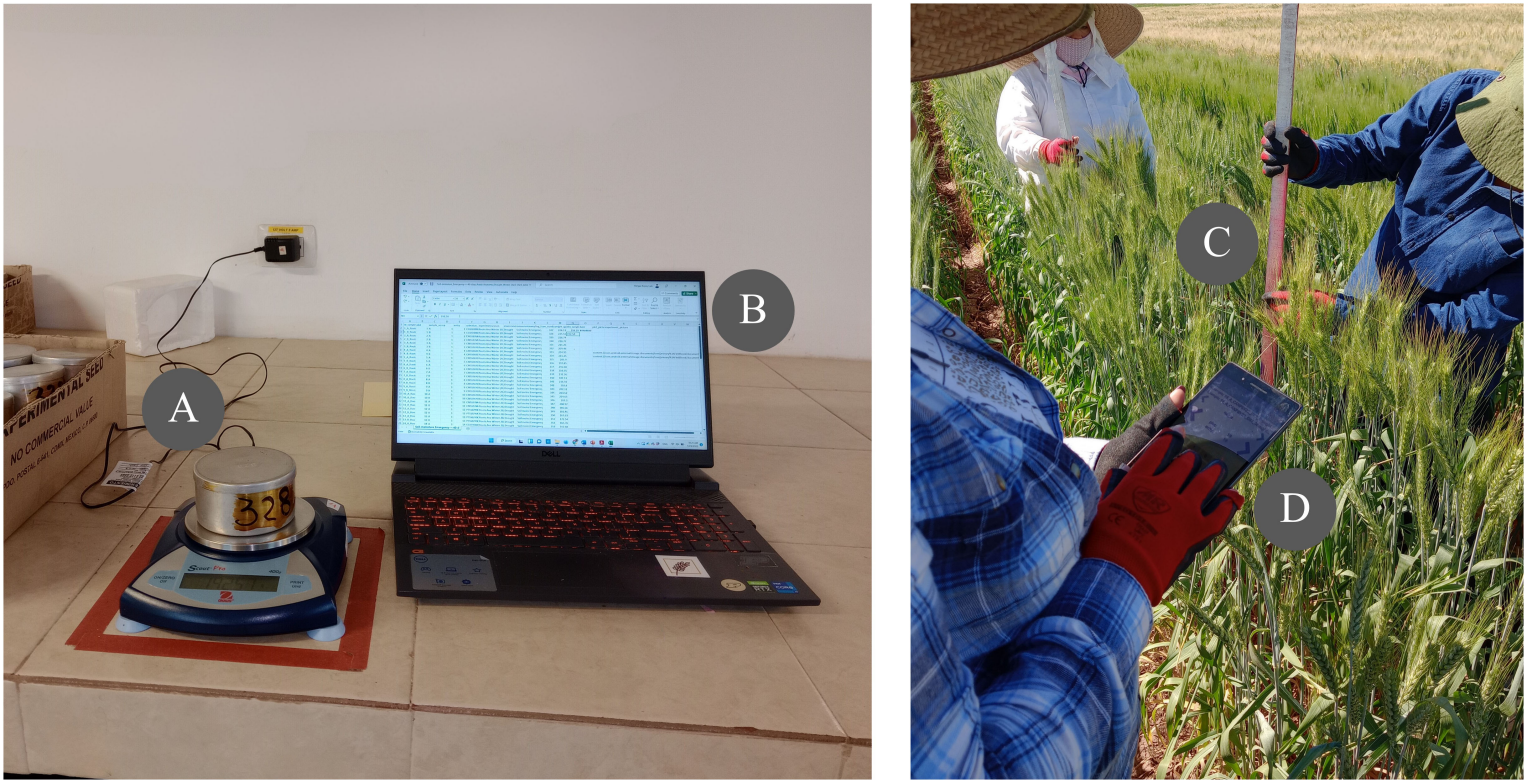
Application of AgTC in wheat experiments. Left: An example of using an AgTC-generated template in laboratory conditions. Here, wet and dry soil weights are measured **(A)** and entered into an AgTC template directly on the computer **(B)**. Right: An example of using an AgTC-generated template in field conditions. In this example, wheat plant height is measured manually **(C)**, and entered digitally in the field via the Field Book application that utilizes an AgTC-generated template **(D)**.

We propose a pipeline for data collection and management using AgTC and AgETL. The first step is to create templates using AgTC. Users can then open the CSV output file to enter data in a standardized fashion (**Figure 1**). If data are acquired in the lab, as would be the case for any sample destructively collected in the field or greenhouse, this can be accomplished using spreadsheet software on a computer (**Figure 3**). Alternatively, the template can be used directly as a field file for the Field Book application to facilitate data collection on-site, such as for data collection in the field, greenhouse, or growth chamber. When data collection is carried out using instruments that come with their own internal data storage system (e.g., the LI-6800 Portable Photosynthesis System), users download resultant files directly. After data acquisition, AgETL functions *Extract* and *Transform* data files from different sources to standardize their formats. From there, researchers can run exploratory data analysis, such as data aggregation, visualization, outlier detection to perform data QC. After data are QC-ed, they are ready for downstream analyses, such as modeling. At this point, it is also advisable to load the QC-ed data into a database using AgETL’s *Load* functions. In addition to enhancing data analysis capabilities via interaction with data analysis dashboards, data are securely stored and easily accessible.

Below, we describe the application of AgTC and AgETL in several test cases. AgTC was utilized in experiments on soybean, rice, and wheat under field and controlled environmental conditions, while AgETL functions were utilized for generating soybean and wheat datasets. In addition to the specific test cases presented here, AgTC has been used on another soybean experiment carried out in Wanatah, Indiana, USA, in 2022 and two more wheat experiments in the same research center at CIMMYT in Mexico during the 2023 field season.

### 3.2 Application of AgTC

Templates created using AgTC were used for data collection for 12 measurements across three species in three experimental settings (**Table 3**). We observed that the new tool enhanced data collection in at least two ways: (1) utilization reduced the total steps required for data collection, and (2) application of AgTC helped improve data file organization.

**Table 3:** Overview of measurements collected using AgTC and/or processed by AgETL from three experimental test cases.

Experimental setting	Measurements	Soybean	Wheat	Rice	Device used for data collection	AgTC	AgETL
Field	Canopy temperature		✔		PMD	✔	✔
	Canopy width	✔			PMD	✔	✔
	Chlorophyll content		✔		PMD	✔	✔
	Hyperspectral reflectance		✔		IMS		✔
	Image of youngest fully expanded trifoliate leaf	✔			PMD	✔	
	Leaf Area Index	✔			IMS		✔
	Normalized difference vegetation index		✔		IMS		✔
	Plant developmental stages dates	✔	✔		PMD	✔	✔
	Plant height	✔	✔		PMD	✔	✔
	Volumetric soil water content	✔			IMS		✔
Growth chamber	Image of a youngest fully expanded leaf			✔	PMD	✔	
	Leaf water potential			✔	PMD	✔	
Lab	Above-ground biomass		✔		PC	✔	✔
	Gravimetric soil water content		✔		PC	✔	✔
	Trifoliate leaf dry weight	✔			PMD	✔	✔
	Yield components	✔	✔		PC	✔	✔

Measurement data were temporarily stored in the Field Book application on personal mobile devices (PMD), in spreadsheets on personal computers (PC), or within internal memory storage (IMS) of individual instruments. The checkmark indicates that measurement was taken in the experiment indicated.

*Reduction in the number of steps for data collection.* In the soybean and rice experimental test cases, SLA was measured at multiple timepoints (five for soybean and two for rice). SLA is computed as the ratio of leaf area and leaf dry weight. Conventionally, leaf area estimates are made using leaf scanners or image-based software, whereby image files are manually reamed utilizing labels found within each image itself prior to image processing. This renaming step is tedious and may be affected by human error. To improve on the conventional method for estimating SLA, AgTC was used to create one template per sampling campaign, which was uploaded as a field file in the Field Book application. Then, using the picture function of Field Book, a photo of the target leaf was taken using tablets in the field. The advantage of this system is that Field Book directly uses the values of the observation identifier column generated by AgTC as the names of the image files. Compared with our traditional method, this saves time by precluding the need to manually rename image files before extracting leaf area values using downstream software such as Easy Leaf Area (Easlon and Bloom, 2014).

Another example where the use of a template generated from AgTC as a field file in the Field Book application helped decrease the number of steps in data collection was for wheat canopy temperature measurements in the field. The conventional method employed by the CIMMYT Wheat Physiology group requires that one researcher takes temperature readings from the crop canopy using a sensor device while another records these values on a paper field form. Then, values are transferred manually to a digital spreadsheet. When using an AgTC-generated template opened either in a mobile device or uploaded as a field file in Field Book, measurements are digitized directly in the field, enhancing the efficiency of data collection and potentially reducing human error involved in converting paper records into digital formats.

*Improvement of data file organization.* For some measurements, AgTC did not minimize the overall number of steps involved in data collection but can improve data management. As an illustration, we describe the process for recording wheat biomass measurements. Conventionally, the data are obtained in a laboratory setting, where the samples are processed, and data are entered directly into electronic files. Using AgTC, the overall process does not change. However, AgTC automatically generates metadata columns and unique identifiers for each observation in the electronic template files without any need to manually copy and paste information across spreadsheets. Additionally, the standardized format for naming template files by AgTC facilitates file organization in storage directories.

### 3.3 Application of AgETL

AgETL was tested successfully for processing data collected from soybean experiments of the 2022 summer season and for data collected from the wheat experiments of winter seasons 2021-2022 and 2022-2023. The *Extract* and *Transform* functions were executed separately from the *Load* functions for these data. Resultant standardized dataframes were loaded into a PostgreSQL database using the Load process after the *Extract* and *Transform* processes.

The main objective of the *Extract* and *Transform* processes of AgETL is to generate dataframes with a standard format and structure using heterogeneously formatted lab- or field-generated data. *Extract* and *Transform* functions that perform the extraction process first take in CSV files from the directory indicated on the configuration file. The next step compares each file’s column names to identify unique and duplicated names. The output of this execution, a list of similar and different column names, helps the user decide which transformations are needed to alter dataframe columns. In the following step, dataframes obtained from the extracted files are concatenated, resulting in a single dataframe where original columns with shared names are combined, and original columns with different names are retained separately. The resulting dataframe is then combined with other files containing additional relevant information (e.g., genotype names) if the user indicated these parameters in the configuration file. Subsequent transformation steps depend on the kinds of alterations the data needs to undergo quality assurance (QA) or enter the *Load* process. At this stage, trait column names can be unified. For example, ‘LAI’, ‘Leaf Area Index’, ‘lai’, and ‘leaf area index’ refer to the same trait yet would be treated differently by database management systems; a transformation to update column names is needed. This and other options for transformations are illustrated in **Supplementary Figure 1** and include dropping undesired columns, creating new columns, updating row values, and updating primary key values.

From our testing of *Extract* and *Transform* processes on the soybean and wheat field experiments, we found AgETL useful for several scenarios. *Scenario 1:* AgETL was applied on data files derived from templates generated using AgTC. For example, we simultaneously processed 42 different canopy temperature files from three treatments and two crop growth seasons in the wheat experiment. In this case, only transformations for updating column names were needed. *Scenario 2:* AgETL was used to process data files derived from lab-collected measurements. From the same wheat experiments, we implemented AgETL on ten biomass files that were collected from samples processed in the lab. After files were joined, multiple column names were updated to unify columns with the same meaning, one column was added to indicate the unit, and several extraneous columns used in intermediate stages of biomass estimation were dropped (e.g., bag dry weight). *Scenario 3:* AgETL was used to process data collected via sensors that had their own unique storage systems. For instance, in the soybean experiment, data files originated from hand-held instruments such as the Campbell HydroSense II and LAI2200C (**Table 3**); in this case, the *Extract* and *Transform* functions of AgETL were used to merge the files into a single dataframe and join genotype names from another data file.

**Figure 4:**
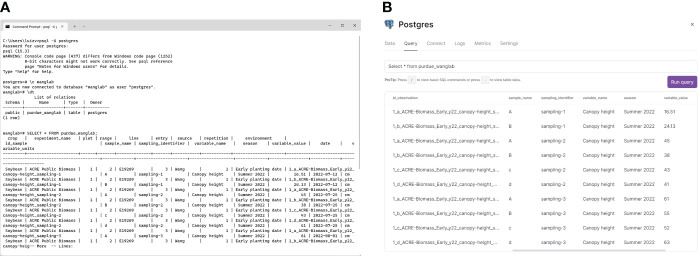
The AgETL *Load* process facilitates the loading of clean data into databases. Shown here are data loaded **(A)** in a PostgreSQL localhost server and **(B)** in Railway, a commercial database cloud service provider.

Standardized, uniformly formatted data files resulting from the execution of *Extract* and *Transform* functions were next uploaded into a PostgreSQL database server to test the utility of AgETL’s *Load* process. The first step is connecting with a PostgreSQL database (either a cloud-hosted instance or a local installation). The configuration file is flexible, allowing the user to write parameters for either of the two options. We successfully tested the database connection in three database scenarios: one was a local instance (**Figure 4A**), and the other two were commercial cloud service providers, *i.e.*, Database as a Service (DBaaS). The two cloud services were Cloud SQL (https://cloud.google.com), offered by Google Cloud Platform (Bisong, 2019), and the Railway PostgreSQL database service (https://railway.app), shown in **Figure 4B**. Instructions for establishing the database connection have been made available in the AgETL GitHub repository. One significant advantage of processing and managing data using AgETL was observed for canopy temperature and SPAD measurements collected weekly during wheat experiments. These data were processed and loaded immediately after the data were gathered. The dashboard automatically reflected updated information in its data visualization features because the database was connected with a real-time data visualization online interface.

After establishing the database connection, users can create the first table by writing the names of the columns and the data types using the PostgreSQL names for native data types on the configuration file. Each table only needs to be created once, and it is possible to create many tables if needed. The recommendation is to create tables based on specific research goals. For instance, data from different experiments that will be used to calibrate crop models should be uploaded to the same table in the database. Once the table exists, the data from the files specified for the user are uploaded.

Moreover, AgETL can add more columns when the table already exists. Additionally, the records in the database can be upgraded by loading the data file with the updated row and having the same primary key. Furthermore, AgETL gives the user access to the four standard SQL actions of database systems called CRUD, which comes from create, read, update, and delete (van den Brink et al., 2007; Krogh, 2018). For that reason, the Jupyter Notebook file has a section with SQL statements, called useful SQL statements, that allows the deletion of a column and a table. Finally, AgETL permits users to write and execute their own SQL statements, like SQL queries, directly from Jupiter Notebook without another extra configuration.

We additionally tested the connection to the DBaaS using R (R Core Team, 2021) and Python, two widely used programming languages in data analysis for research (Fahad and Yahya, 2018; Rahmany et al., 2020). We successfully connected R with the two DBaaS providers using the R packages, DBI (R Special Interest Group on Databases (R-SIG-DB) et al., 2022), and odbc (Hester and Wickham, 2023). Finally, the RPostgreSQL R package was used to access the PostgreSQL database. For testing with Python, the PostgreSQL connection was successfully created using the Psycopg2 library (https://www.psycopg.org).

From our experience developing and testing AgTC and AgETL in the soybean, wheat, and field experiments, we have five suggestions to best leverage their use in small to mid-sized research groups: (1) Users who collect data should be the ones that create their own template files with AgTC, and (2) these templates should be saved on a centralized repository. The creation of multiple nested folders should be avoided since the template files can be sorted by their names, which are automatically generated in a structured, consistent, and meaningful way. For the *Extract* and *Transform* process using AgETL, it may be best to (3) appoint a single responsible person to handle these steps, while (4) individual researchers execute the QA/QC themselves on the files generated from the *Extract* and *Transform* steps, following and documenting steps appropriate for their own research project. Finally, (5) an appointed individual in the research group (or IT specialist collaborating with the research group) handles uploading clean, QA/QC-ed files to the database using AgETL’s *Load* process. This should occur on a regular basis that is sensible for the research group, *e.g.*, after each field season, to keep the database up-to-date.

### 3.4 Areas for improvement

We have identified several areas for future improvements to AgTC and AgETL. Even though AgTC templates allow the collection of multiple variables at the same time (e.g., as would occur if plant height and canopy width are collected with the same AgTC-generated template), multivariable files used as input in AgETL need to be processed as separate steps during the *Extract* and *Transform* stages mainly because the observation ID need to be updated. This was to make the configuration file simpler for the user, such that they do not need to specify many parameters. Streamlining these processing steps into a single step for multivariable files could be an improvement in an updated version of the tool. Additionally, documentation to implement ETL steps using functions on workflow management platforms such as Apache Airflow would help to automate AgETL. Finally, documentation to use both AgTC and AgETL in command-line mode could enable the implementation of these tools as part of a larger workflow, which may be particularly useful for moderate to large-sized laboratories.

## 4 Conclusion

We have developed two tools to address observed challenges in data collection, processing, and management in plant science research called AgTC and AgETL. These tools are simple to use and do not require experience in programming. Additionally, they are adaptable for data collection and data processing in the field or the lab and are agnostic to crop species, experimental design, and scale. The templates generated by AgTC can be used for data collection in the field or the lab and can reduce the number of steps required for data collection and improve data file organization. AgETL enables the extraction of information from multiple files from diverse sources and measurements and merges them into the same data table. This tool facilitates loading and wrangling data on a local host or using a database cloud service, which can be readily managed by appointed individuals within research labs or by collaborating with institutional IT support. Finally, we have developed user documentation, with English and Spanish versions available in the README section of the AgTC and AgETL GitHub repositories.

## Data availability statement

The source code for AgTC and AgETL can be found here: https://github.com/DS4Ag/AgETL, https://github.com/DS4Ag/AgTC.

## Author contributions

LV-R: Conceptualization, Methodology, Software, Writing – original draft, Writing – review & editing. T-CT: Methodology, Writing – review & editing. KR: Resources, Writing – review & editing. MR: Resources, Funding acquisition, Project administration, Writing – review & editing. DW: Funding acquisition, Resources, Writing – review & editing, Conceptualization, Supervision, Writing – original draft.
