# Supplementary material for: AgTC and AgETL: open-source tools to enhance data collection and management for plant science research
Source: Front Plant Sci. 2024 Feb 21;15:1265073. doi: 10.3389/fpls.2024.1265073 (PMC10915008; doi:10.3389/fpls.2024.1265073)
Supplement: Supplementary file 1 [file DataSheet_1.pdf]

Supplementary Material

AgTC and AgETL: Open-source tools to enhance data collection and management for plant science research

Luis Vargas-Rojas[1], To-Chia Ting[1], Katherine Rainey[1], Matthew Reynolds[2], Diane R. Wang[1]*

[1]Department of Agronomy, Purdue University, West Lafayette, IN 47906, USA

[2]International Maize and Wheat Improvement Center (CIMMYT), Texcoco, 56237, Mexico

*** Correspondence:**
Diane R. Wang
drwang@purdue.edu

1. Supplementary Figures and Tables

1.1 Supplementary Figure

1.2 Supplementary Table

Step 1. Concatenate data files and join additional information

id_observation	sample -name	plot	experiment	season	trait	sampling -id	Height (cm)	date	notes
1_a_Biomass_y22_height_s-1	a	1	Biomass	y22	height	s-1	16.51	7/25/2022	
1_b_Biomass_y22_height_s-1	b	1	Biomass	y22	height	s-1	24.13	7/25/2022	
1_a_Biomass_y22_height_s-1	a	2	Biomass	y22	height	s-1	39.37	7/25/2022	
1_b_Biomass_y22_height_s-1	b	2	Biomass	y22	height	s-1	26.67	7/25/2022	

id_observation	plot	sample _name	experiment	sampling-id	trait	season	LAI	DATE	TIME
1_a_Biomass_y22_LAI_s-1	1	A	Biomass	s-1	LAI	y22	2.722	7/25/2022	7:26 AM
1_b_Biomass_y22_LAI_s-1	1	B	Biomass	s-1	LAI	y22	2.165	7/25/2022	7:27 AM
1_a_Biomass_y22_LAI_s-1	2	A	Biomass	s-1	LAI	y22	1.105	7/25/2022	7:28 AM
1_b_Biomass_y22_LAI_s-1	2	B	Biomass	s-1	LAI	y22	1.906	7/25/2022	7:29 AM

plot	entry	line
1	3	E19269
2	24	U15-606207

id_observation	plot	sample -name	experiment	season	sampling -id	trait	Height (cm)	LAI	date	TIME	notes	line
1_a_Biomass_y22_height_s-1	1	a	Biomass	y22	s-1	height	16.51		7/25/2022			E19269
1_b_Biomass_y22_height_s-1	1	b	Biomass	y22	s-1	height	24.13		7/25/2022			E19269
1_a_Biomass_y22_height_s-1	2	a	Biomass	y22	s-1	height	39.37		7/25/2022			U15-606207
1_b_Biomass_y22_height_s-1	2	b	Biomass	y22	s-1	height	26.67		7/25/2022			U15-606207
1_a_Biomass_y22_LAI_s-1	1	A	Biomass	y22	s-1	LAI		2.722	7/25/2022	7:26 AM		E19269
1_b_Biomass_y22_LAI_s-1	1	B	Biomass	y22	s-1	LAI		2.165	7/25/2022	7:27 AM		E19269
1_a_Biomass_y22_LAI_s-1	2	A	Biomass	y22	s-1	LAI		1.105	7/25/2022	7:28 AM		U15-606207
1_b_Biomass_y22_LAI_s-1	2	B	Biomass	y22	s-1	LAI		1.906	7/25/2022	7:29 AM		U15-606207

Step 2. Drop undesired columns

id_observation	sample -name	plot	experiment	season	sampling -id	trait	Height (cm)	LAI	date	TIME	notes	line
1_a_Biomass_y22_height_s-1	a	1	Biomass	y22	s-1	height	16.51		7/25/2022			E19269
1_b_Biomass_y22_height_s-1	b	1	Biomass	y22	s-1	height	24.13		7/25/2022			E19269
1_a_Biomass_y22_height_s-1	a	2	Biomass	y22	s-1	height	39.37		7/25/2022			U15-606207
1_b_Biomass_y22_height_s-1	b	2	Biomass	y22	s-1	height	26.67		7/25/2022			U15-606207
1_a_Biomass_y22_LAI_s-1	A	1	Biomass	y22	s-1	LAI		2.722	7/25/2022	7:26 AM		E19269
1_b_Biomass_y22_LAI_s-1	B	1	Biomass	y22	s-1	LAI		2.165	7/25/2022	7:27 AM		E19269
1_a_Biomass_y22_LAI_s-1	A	2	Biomass	y22	s-1	LAI		1.105	7/25/2022	7:28 AM		U15-606207
1_b_Biomass_y22_LAI_s-1	B	2	Biomass	y22	s-1	LAI		1.906	7/25/2022	7:29 AM		U15-606207

→

id_observation	sample -name	plot	experiment	season	sampling -id	trait	Height (cm)	LAI	date	line
1_a_Biomass_y22_height_s-1	a	1	Biomass	y22	s-1	height	16.51		7/25/2022	E19269
1_b_Biomass_y22_height_s-2	b	1	Biomass	y22	s-1	height	24.13		7/25/2022	E19269
1_a_Biomass_y22_height_s-1	a	2	Biomass	y22	s-1	height	39.37		7/25/2022	U15-606207
1_b_Biomass_y22_height_s-2	b	2	Biomass	y22	s-1	height	26.67		7/25/2022	U15-606207
1_a_Biomass_y22_LAI_s-1	A	1	Biomass	y22	s-1	LAI		2.722	7/25/2022	E19269
1_b_Biomass_y22_LAI_s-1	B	1	Biomass	y22	s-1	LAI		2.165	7/25/2022	E19269
1_a_Biomass_y22_LAI_s-1	A	2	Biomass	y22	s-1	LAI		1.105	7/25/2022	U15-606207
1_b_Biomass_y22_LAI_s-1	B	2	Biomass	y22	s-1	LAI		1.906	7/25/2022	U15-606207

Step 3. Update column names

id_observation	sample -name	plot	experiment	season	sampling -id	trait	Height (cm)	LAI	date	line
1_a_Biomass_y22_height_s-1	a	1	Biomass	y22	s-1	height	16.51		7/25/2022	E19269
1_b_Biomass_y22_height_s-1	b	1	Biomass	y22	s-1	height	24.13		7/25/2022	E19269
1_a_Biomass_y22_height_s-1	a	2	Biomass	y22	s-1	height	39.37		7/25/2022	U15-606207
1_b_Biomass_y22_height_s-1	b	2	Biomass	y22	s-1	height	26.67		7/25/2022	U15-606207
1_a_Biomass_y22_LAI_s-1	A	1	Biomass	y22	s-1	LAI		2.722	7/25/2022	E19269
1_b_Biomass_y22_LAI_s-1	B	1	Biomass	y22	s-1	LAI		2.165	7/25/2022	E19269
1_a_Biomass_y22_LAI_s-1	A	2	Biomass	y22	s-1	LAI		1.105	7/25/2022	U15-606207
1_b_Biomass_y22_LAI_s-1	B	2	Biomass	y22	s-1	LAI		1.906	7/25/2022	U15-606207

→

id_observation	sample-name	plot	experiment	season	sampling-id	trait	value	date	line
1_a_Biomass_y22_height_s-1	a	1	Biomass	y22	s-1	height	16.51	7/25/2022	E19269
1_b_Biomass_y22_height_s-1	b	1	Biomass	y22	s-1	height	24.13	7/25/2022	E19269
1_a_Biomass_y22_height_s-1	a	2	Biomass	y22	s-1	height	39.37	7/25/2022	U15-606207
1_b_Biomass_y22_height_s-1	b	2	Biomass	y22	s-1	height	26.67	7/25/2022	U15-606207
1_a_Biomass_y22_LAI_s-1	A	1	Biomass	y22	s-1	LAI	2.722	7/25/2022	E19269
1_b_Biomass_y22_LAI_s-1	B	1	Biomass	y22	s-1	LAI	2.165	7/25/2022	E19269
1_a_Biomass_y22_LAI_s-1	A	2	Biomass	y22	s-1	LAI	1.105	7/25/2022	U15-606207
1_b_Biomass_y22_LAI_s-1	B	2	Biomass	y22	s-1	LAI	1.906	7/25/2022	U15-606207

Step 4. Insert new columns

id_observation	sample -name	plot	experiment	season	sampling -id	trait	value	date	line
1_a_Biomass_y22_height_s-1	a	1	Biomass	y22	s-1	height	16.51	7/25/2022	E19269
1_b_Biomass_y22_height_s-1	b	1	Biomass	y22	s-1	height	24.13	7/25/2022	E19269
1_a_Biomass_y22_height_s-1	a	2	Biomass	y22	s-1	height	39.37	7/25/2022	U15-606207
1_b_Biomass_y22_height_s-1	b	2	Biomass	y22	s-1	height	26.67	7/25/2022	U15-606207
1_a_Biomass_y22_LAI_s-1	A	1	Biomass	y22	s-1	LAI	2.722	7/25/2022	E19269
1_b_Biomass_y22_LAI_s-1	B	1	Biomass	y22	s-1	LAI	2.165	7/25/2022	E19269
1_a_Biomass_y22_LAI_s-1	A	2	Biomass	y22	s-1	LAI	1.105	7/25/2022	U15-606207
1_b_Biomass_y22_LAI_s-1	B	2	Biomass	y22	s-1	LAI	1.906	7/25/2022	U15-606207

→

id_observation	sample -name	plot	experiment	season	sampling -id	trait	value	units	date	line
1_a_Biomass_y22_height_s-1	a	1	Biomass	y22	s-1	height	16.51	cm	7/25/2022	E19269
1_b_Biomass_y22_height_s-1	b	1	Biomass	y22	s-1	height	24.13	cm	7/25/2022	E19269
1_a_Biomass_y22_height_s-1	a	2	Biomass	y22	s-1	height	39.37	cm	7/25/2022	U15-606207
1_b_Biomass_y22_height_s-1	b	2	Biomass	y22	s-1	height	26.67	cm	7/25/2022	U15-606207
1_a_Biomass_y22_LAI_s-1	A	1	Biomass	y22	s-1	LAI	2.722	LAI	7/25/2022	E19269
1_b_Biomass_y22_LAI_s-1	B	1	Biomass	y22	s-1	LAI	2.165	LAI	7/25/2022	E19269
1_a_Biomass_y22_LAI_s-1	A	2	Biomass	y22	s-1	LAI	1.105	LAI	7/25/2022	U15-606207
1_b_Biomass_y22_LAI_s-1	B	2	Biomass	y22	s-1	LAI	1.906	LAI	7/25/2022	U15-606207

Step 5. Update row values

id_observation	sample -name	plot	experiment	season	sampling -id	trait	value	units	date	line
1_a_Biomass_y22_height_s-1	a	1	Biomass	y22	s-1	height	16.51	cm	7/25/2022	E19269
1_b_Biomass_y22_height_s-2	b	1	Biomass	y22	s-1	height	24.13	cm	7/25/2022	E19269
1_a_Biomass_y22_height_s-1	a	2	Biomass	y22	s-1	height	39.37	cm	7/25/2022	U15-606207
1_b_Biomass_y22_height_s-2	b	2	Biomass	y22	s-1	height	26.67	cm	7/25/2022	U15-606207
1_a_Biomass_y22_LAI_s-1	A	1	Biomass	y22	s-2	LAI	2.722	LAI	7/25/2022	E19269
1_b_Biomass_y22_LAI_s-2	B	1	Biomass	y22	s-2	LAI	2.165	LAI	7/25/2022	E19269
1_a_Biomass_y22_LAI_s-1	A	2	Biomass	y22	s-2	LAI	1.105	LAI	7/25/2022	U15-606207
1_b_Biomass_y22_LAI_s-2	B	2	Biomass	y22	s-2	LAI	1.906	LAI	7/25/2022	U15-606207

→

id_observation	sample-name	plot	experiment	season	sampling-id	trait	value	units	date	line
1_a_Biomass_y22_height_s-1	a	1	Biomass	2022	s-1	Canopy height	16.51	cm	7/25/2022	E19269
1_b_Biomass_y22_height_s-2	b	1	Biomass	2022	s-1	Canopy height	24.13	cm	7/25/2022	E19269
1_a_Biomass_y22_height_s-1	a	2	Biomass	2022	s-1	Canopy height	39.37	cm	7/25/2022	U15-606207
1_b_Biomass_y22_height_s-2	b	2	Biomass	2022	s-1	Canopy height	26.67	cm	7/25/2022	U15-606207
1_a_Biomass_y22_LAI_s-1	a	1	Biomass	2022	s-2	LAI	2.722	LAI	7/25/2022	E19269
1_b_Biomass_y22_LAI_s-2	b	1	Biomass	2022	s-2	LAI	2.165	LAI	7/25/2022	E19269
1_a_Biomass_y22_LAI_s-1	a	2	Biomass	2022	s-2	LAI	1.105	LAI	7/25/2022	U15-606207
1_b_Biomass_y22_LAI_s-2	b	2	Biomass	2022	s-2	LAI	1.906	LAI	7/25/2022	U15-606207

Step 6. Update primary key values

id_observation	sample-name	plot	experiment	season	sampling-id	trait	value	units	date	line
1_a_Biomass_y22_height_s-1	a	1	Biomass	2022	s-1	Canopy height	16.51	cm	7/25/2022	E19269
1_b_Biomass_y22_height_s-1	b	1	Biomass	2022	s-1	Canopy height	24.13	cm	7/25/2022	E19269
1_a_Biomass_y22_height_s-1	a	2	Biomass	2022	s-1	Canopy height	39.37	cm	7/25/2022	U15-606207
1_b_Biomass_y22_height_s-1	b	2	Biomass	2022	s-1	Canopy height	26.67	cm	7/25/2022	U15-606207
1_a_Biomass_y22_LAI_s-1	a	1	Biomass	2022	s-1	LAI	2.722	LAI	7/25/2022	E19269
1_b_Biomass_y22_LAI_s-1	b	1	Biomass	2022	s-1	LAI	2.165	LAI	7/25/2022	E19269
1_a_Biomass_y22_LAI_s-1	a	2	Biomass	2022	s-1	LAI	1.105	LAI	7/25/2022	U15-606207
1_b_Biomass_y22_LAI_s-1	b	2	Biomass	2022	s-1	LAI	1.906	LAI	7/25/2022	U15-606207

→

id_observation	sample -name	plot	experiment	season	sampling -id	trait	value	units	date	line
1_a_Biomass_y22_Canopy-height_s-1	a	1	Biomass	2022	s-1	Canopy height	16.51	cm	7/25/2022	E19269
1_b_Biomass_y22_Canopy-height_s-1	b	1	Biomass	2022	s-1	Canopy height	24.13	cm	7/25/2022	E19269
1_a_Biomass_y22_Canopy-height_s-1	a	2	Biomass	2022	s-1	Canopy height	39.37	cm	7/25/2022	U15-606207
1_b_Biomass_y22_Canopy-height_s-1	b	2	Biomass	2022	s-1	Canopy height	26.67	cm	7/25/2022	U15-606207
1_a_Biomass_y22_LAI_s-1	a	1	Biomass	2022	s-1	LAI	2.722	LAI	7/25/2022	E19269
1_b_Biomass_y22_LAI_s-1	b	1	Biomass	2022	s-1	LAI	2.165	LAI	7/25/2022	E19269
1_a_Biomass_y22_LAI_s-1	a	2	Biomass	2022	s-1	LAI	1.105	LAI	7/25/2022	U15-606207
1_b_Biomass_y22_LAI_s-1	b	2	Biomass	2022	s-1	LAI	1.906	LAI	7/25/2022	U15-606207

Supplementary Figure 1. Transformations made in the Extract and Transforms steps of the AgETL tool.

1.1 Supplementary tables

Supplementary table 1. Dates of two critical growth stages (GS) (Zadoks et al., 1974), seedling emergence (SE) and physiological maturity (PM), of the wheat panel under well-watered (WW), drought (DR), and high temperature (HT) environments.

	Growth season 2022		Growth season 2023	
Environment	SE (GS 10)	PM (GS 87)	SE	PM
WW	12/1/2021	*	11/30/2022	04/06/2022 - 04/19/2023
DR	12/9/2021	3/15/2022 - 04/01/2022	12/9/2021	03/25/2023 - 04/04/2023
HT	3/11/2022	05/18/2022 - 05/26/2022	**	**

* Data were collected only from GS10 to GS54; ** the treatment was not planted during this season.
